# Investigation of the histopathological level of Ki-67, caspase-3 expressions of the effects of hesperidin on wound healing in the rat esophagus

**DOI:** 10.1590/acb381723

**Published:** 2023-04-21

**Authors:** Cemalettin Durgun, Gülsen Kirman, Engin Deveci

**Affiliations:** 1 Diyarbakır Memorial Hospital – Department of General Surgery – Diyarbakır, Turkey.; 2 Siirt University – Faculty of Veterinary Medicine – Department of Histology and Embryology – Siirt, Turkey.; 3 Dicle Üniversitesi – Medical Faculty – Department of Histology and Embryology – Diyarbakır, Turkey.

**Keywords:** Esophagus, Burns, Apoptosis, Ki-67 antigen, Rats

## Abstract

**Purpose:**

The effects of hesperidin application on the wound caused by esophageal burns were investigated in this study.

**Methods:**

Wistar albino rats were divided into three groups: Control group: only 1 mL of 0.09% NaCl was administered i.p. for 28 days; Burn group: An alkaline esophageal burn model was created with 0.2 mL of 25% NaOH orally by gavage—1 mL of 0.09% NaCl was administered i.p. for 28 days; Burn+Hesperidin group: 1 mL of 50 mL/kg of hesperidin was given i.p. for 28 days to rats after burn injury. Blood samples were collected for biochemical analysis. Esophagus samples were processed for histochemical staining and immunohistochemistry.

**Results:**

Malondialdehyde (MDA) and myeloperoxidase (MPO) levels were significantly increased in Burn group. Glutathione (GSH) content and histological scores of epithelialization, collagen formation, neovascularization was decreased. After hesperidin treatment, these values were significantly improved in the Burn+Hesperidin group. In the Burn group, epithelial cells and muscular layers were degenerated. Hesperidin treatment restored these pathologies in Burn+Hesperidin group. Ki-67 and caspase-3 expressions were mainly negative in control group; however, the expression was increased in the Burn group. In the Burn+Hesperidin group, Ki-67 and caspase-3 immune activities were reduced.

**Conclusions:**

Hesperidin dosage and application methods can be developed as an alternative treatment for burn healing and treatment.

## Introduction

Corrosive substances are frequently used in daily life, and their accidental ingestion is one of the common reasons for applying to the emergency department all over the world, especially in childhood. Exposure to these substances poses serious problems in various age groups^1^. Children under the age of 5 make up 80% of the cases. Ingestion of corrosive substances can cause serious damage to the esophagus and stomach. Damage depends on the corrosive properties of the ingested substance, its concentration, amount, physical form, and contact time with the mucosa^2^. Corrosive esophageal burns can occur with substances such as sulfuric and hydrochloric acid, as well as the ingestion of strong alkalis such as (75–90%) potassium hydroxide, sodium hydroxide (NaOH) and sodium hypochlorite^3^. Corrosive burns are common causes of mortality and morbidity that can progress from stenosis to perforation in the upper gastrointestinal tract. The risk of developing esophageal carcinoma after corrosive esophagitis is 1,000–3,000 times higher than in the normal population^4^.

Hesperidin is an important flavonoid, and its chemical formula is C_28_H_34_O_15_. Hesperidin has a low molecular weight and belongs to the flavanone class of flavonoids^5^. Hesperidin (hesperetin-7-O-rutinoside) is the β-glycoside form of the flavanone hesperidin and is found almost exclusively in citrus fruits, especially oranges. A glycoside is a molecule consisting of a sugar and a non-sugar group, called an aglycone. Hesperidin is a good free radical chelator. Other researchers have studied the antioxidant activity of hesperidin and its radical scavenging properties using various assay systems. Hesperidin has been reported to reduce superoxide ions in electron transfer, as well as in vitro compatible proton transfer reaction. Apart from the antioxidant effect of hesperidin, its effects on the vascular system (it regulates capillary permeability), anti-inflammatory effect, antioxidant effect, effect on enzymes, antimicrobial activity (antibacterial, antifungal, antiviral), anticarcinogenic activity, inhibition of cell aggregation, antiallergic effect, ultraviolet protection activity are known^5^^-^ 7.

It has been stated that the protective effect of hesperidin, which is included in citrus peel extracts, on the gastrointestinal tract is more remarkable for higher biopolymer concentrations^8^. Similarly, chachiensis and grapefruit citrus peel flavonoid extracts have been shown to have positive effects on gut microecology, as evidenced by a significant increase in the relative abundance of *Bifidobacterium* spp. model. Li et al. particularly emphasized the biological function of citrus peel flavonoid extracts as prebiotic agents, drawing attention to their potential use in food and biomedical applications^9^. The hepatoprotective/gastroprotective effects of aqueous and butanol citrus peel extracts and hesperidin were experimentally evaluated in rat models of ulcer and hepatotoxicity. Abou Baker et al. reported that hesperidin showed the best hepatoprotective, antioxidant, anti-inflammatory and gastroprotective effects, followed by butanol and aqueous citrus peel extracts^10^. In another gastrointestinal study, oral administration of hesperidin was shown to effectively alleviate rat postoperative ileus through inhibition of inflammatory responses and stimulation of Ca(2+)-dependent myosin light chain kinase phosphorylation^11^.

Ki-67 is a popular marker used to detect cellular proliferation during burn injury. It is a protein related to cell cycle and synthesized by all proliferation cells^12^. Caspase-3 is a member of the caspase family that involves in initiating the cell death. Caspase-3 is effector protease that cleaves many targets. During burn injury, both cell death and cell proliferation process are initiated to maintain cellular homeostasis^7^^,^ 13.

The aim of our study is to examine the effectiveness of hesperidin, which has antioxidant capacity, in the wound healing process in esophageal burn induction.

## Methods

### Animals and surgical procedure

All animal experimentation with ethical approval was done according to Local Ethical Committee of Animal Experiments of Siirt University, Turkey. Rats were obtained from Animal Center of Dicle University. Male Wistar albino rats (n = 30), 3 to 4 months old, weighing 180 to 240 g were used. Experimental animals have unlimited access to water and food, and they were kept under control in an environment of 12 h daytime/12 h dark, 8:00 am to 8:00 pm, at 23 ± 2 °C. Animals were categorized into three groups (10 rats per group).

### Esophageal burn induction

Rats were fasted for 12 h for burn induction. A catheter (catalog no: brn02183, Easyflow, Istanbul, Turkey) was used to measure distance from the mouth to the stomach (cardia) region under general anesthesia. The balloon of the silicone urinary catheter was inflated in the stomach and the foley catheter was withdrawn to prevent the passage of 0.2 mL 0.9% NaCl (catalog no: S9888, Sigma Aldrich, Germany) into the stomach. To prevent aspiration, the animals were held at a 90° angle; 0.2 mL of 0.9% NaOH (catalog No: S5881, Sigma Aldrich, Germany) was administered from the proximal part of the balloon to the esophageal lumen, left for 60 s and then aspirated.

### Experimental groups

Control group: 1 mL of 0.09% NaCl was administered i.p. for 28 days to rats without a burn model. Burn group: After esophageal burn induction, 1 mL of 0.09% NaCl was administered i.p. for 28 days to the rats in which the burn model was created. Burn+Hesperidin group: After esophageal burn induction, 1 mL of 50 mL/kg of hesperidin (catalog no: PHR1794, Sigma Aldrich, Germany) was given i.p. for 28 days to rats with a burn model. At the end of experiment all rats were sacrificed.

### Biochemical analyses

Tissues of the esophagus samples were collected for further biochemical analysis. Tissues were placed into gel separator and centrifuged for 5 min at 1,550 g. The supernatant was removed and placed in polypropylene plastic tubes. The tubes were properly labeled with the appropriate sample name and type. Samples were taken and stored at −80 °C for the determination of the malondialdehyde (MDA), glutathione (GSH), and myeloperoxidase (MPO). MDA levels were determined using the double heating method of Draper and Hadley^14^. MDA values were expressed as nmol/g of wet tissue. The GSH activity was measured by the method of Paglia and Valentine^15^. It was observed that the increase in MDA value in the Burn group increased lipid peroxidation and induced cell degeneration, and MPO and GSH values developed in accordance with MDA. As a result, it was observed that increased inflammation in the Burn group caused cell apoptosis. Data were expressed as U/g protein. MPO activity in tissues was measured by a procedure similar to that described by Hillegass et al.^16^. MPO is expressed as U/g of tissue.

### Histopathological analysis

Tissues of the esophagus sections were obtained for histopathological analysis and were fixed in 10% buffered formalin, dehydrated in ethanol (50% to 100%), purified in xylene, and embedded in paraffin. Sections (4–5 mm thick) were cut and stained with hematoxylin and eosin (H&E). The sections were studied to assess the pathological changes in the tissues of the esophagus^17^.

### Immunohistochemical analysis

Formaldehyde-fixed tissue was embedded in paraffin wax for further immunohistochemical examination. Sections were deparaffinized in xylene and passed through descending alcohol series. The antigen retrieval process was performed in citrate buffer solution (pH 6.0) for 15 min in a microwave oven at 700 W. Sections were allowed to cool at room temperature for 30 min and washed twice in phosphate buffered saline (PBS) for 5 min. Endogenous peroxidase blockage was performed in a 3% hydrogen peroxide solution for 7 min. The washed samples were incubated in Ultra V block for 8 min. Blocking solution was removed from the sections and allowed to incubate overnight at +4 °C with primary antibodies epidermal growth factor, (catalog no: ab9695, Abcam, Cambridge, UK) and fibroblast growth factor (catalog no: ab92337, Abcam, Cambridge, UK). After washing the sections in PBS, secondary antibody was applied for 20 min. The sections were washed in PBS for 2×5 min and then exposed to streptavidin-peroxidase for 20 min. Sections washed with PBS were allowed to react with 3,3′-diaminobenzidine chromogen. Counterstaining with hematoxylin was applied and after washing, the preparations were mounted. Sections were examined under a light microscope (Zeiss Imager A2, Jena, Germany)^18^^-^ 20.

### Statistical analysis

Data were analyzed by the IBM SPSS 25.0 software (IBM, Armonk, New York, USA). Shapiro–Wilk test was used for data distribution analysis. Kruskal–Wallis test was used for multiple comparisons with post-hoc Bonferroni test. P < 0.05 was used as the significance level.

## Results

**Figure 1:**
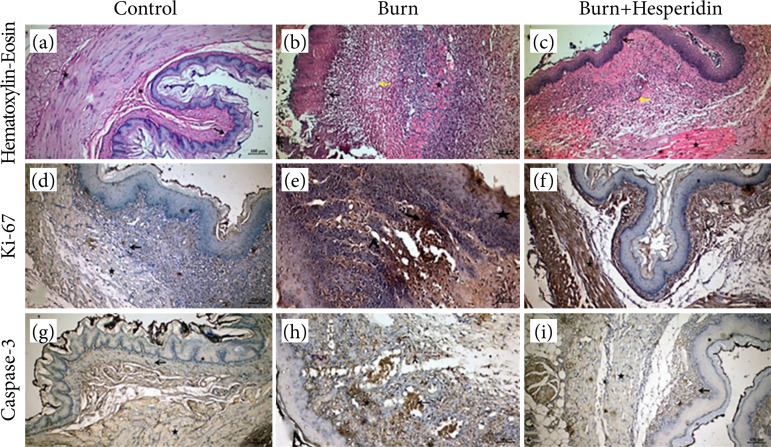
Demonstration of H&E **(a–c)**, Ki-67 **(d–f)**, and caspase-3 **(g–i)** findings in all groups. **(a)** Intense keratinized epithelium (arrowhead) and the basement regular membrane with normal the connective tissue cells (asterisk) and the blood vessels (star); **(b)** Degenerations and nuclei loss (arrowhead), increased apoptosis in the basement membrane (black arrow) and intense leukocyte infiltration (yellow star), vascular dilatation and congestion (asterisk), and degeneration in the muscle layer (star); **(c)** Regular epithelium was regular (arrowhead) with regular basement membrane (black arrow), and decreased leukocytes (yellow arrow); **(d)** Negative ki-67 expression in the epithelial layer (asterisk), lamina propria (arrow), and muscularis mucosa (star); **(e)** Intense ki-67 expression in germinative cells (asterisk), inflammatory cells (arrow), endothelial cells (arrowhead), and lamina propria (star); **(f)** Positive ki-67 expression in the epithelial layer (asterisk), some inflammatory cells (arrow), vascular structures (arrowhead), and muscle bundles (star); **(g)** Negative caspase-3 expression in the esophageal epithelium (asterisk), lamina propria (arrow), and muscle layer (star); **(h)** Intense caspase-3 expression in the lamina propria (star), inflammatory cells (arrow), and connective tissue cells (arrowhead); **(i)** Negative caspase-3 expression in the epithelial layer (asterisk), lamina propria (arrow), and muscle layer (star). Scale bar: 100 μm, Magnification: 10×.

Statistical analysis of biochemical and histochemical parameters was shown in Table 1. MDA and MPO levels and caspase-3 expression were higher in the Burn group than in the Control group. Histological scores of epithelialization, collagen formation, neovascularization and Ki-67 expression were nonsignificantly lower in in the Burn group than in the Control group. GSH content was the significantly lower in the Burn group compared to Control group. After hesperidin treatment, MDA and MPO levels were decreased in Burn+Hesperidin group compared to Burn group, and this decrease was statistically significant. Similarly, GSH content was statistically increased in Burn+Hesperidin group compared to Burn group. Histological scores of epithelialization, collagen formation, neovascularization, and Ki-67 expression were increased in the Burn+Hesperidin group compared to Burn group. Caspase-3 expression was significantly reduced in Burn+Hesperidin group compared to Burn group. Graphical illustration of Table 1 is shown in Fig. 1.

**Table 1:** Biochemical and histological parameters of Control, Burn and Burn+Hesperidin groups.

Parameter	Groups	n	Median (IQR)	P value
MDA	Control	10	25.05 (23.57)	*p = 0.001 **p < 0.043
	Burn	10	49.16 (50.23)	
	Burn+Hesperidin	10	34.48 (38.84)	
GSH	Control	10	1.43 (1.36)	*p < 0.001 **p < 0.001
	Burn	10	0.53 (0.67)	
	Burn+Hesperidin	10	1.42 (1.56)	
MPO	Control	10	3.53 (2.78)	*p < 0.001 **p < 0.001
	Burn	10	8.98 (4.50)	
	Burn+Hesperidin	10	5.52 (5.93)	
Epithelialization	Control	10	2.0 (2.0)	*p = 0.179 **p = 0.003
	Burn	10	0.0 (1.0)	
	Burn+Hesperidin	10	2.0 (2.0)	
Collagen formation	Control	10	2.0 (2.0)	*p = 0.215 **p = 0.031
	Burn	10	0.0 (1.0)	
	Burn+Hesperidin	10	2.0 (2.0)	
Neovascularization	Control	10	3.0 (2.0)	*p = 0.552 **p < 0.001
	Burn	10	0.5 (1.0)	
	Burn+Hesperidin	10	2.5 (1.5)	
Ki-67 expression	Control	10	1.5 (1.5)	*p = 0.239 **p = 0.011
	Burn	10	1.0 (1.0)	
	Burn+Hesperidin	10	2.0 (2.0)	
Caspase-3 expression	Control	10	1.0 (1.0)	*p = 0.196 **p = 0.002
	Burn	10	3.0 (2.0)	
	Burn+Hesperidin	10	1.5 (2.0)	

* Control vs Burn;

** Burn vs Burn+Hesperidin.

**–**

Normal histology of esophagus was observed in Control group. In Burn group, epithelial degeneration, vascular dilatation, and inflammation were observed. Hesperidin treatment alleviates the pathology after esophageal burn induction in Burn+Hesperidin group (Fig. 1c, H&E). Ki-67 and caspase-3 immune expression increased in Burn group, but its level was decreased after hesperidin treatment (Fig. 1e and h, Ki-67 and caspase-3 immune staining). The protective effect of hesperidin showed a significant improvement especially against inflammation and apoptosis.

## Discussion

Wound healing is a complex process which is initiated in response to tissue injury. The healing process requires many cells to cell interactions during inflammation, proliferation, and remodeling. Reactive oxygen species are produced during the tissue injury^21^. Gastrointestinal burns caused by corrosive substances are progressive and cause corrosive lesions in the esophagus. These burns in the esophagus are difficult to treat surgically. The use of alkaline substances causes inflammation, necrosis, and damage to the lipoprotein membranes in the esophagus. Histological layers of the esophagus (mucous, submucosa and muscle layers) cause perforations within seconds after contact with alkaline agents. Alkaline substances spread rapidly to the body tissues because the hydroxy radical they contain binds to the free hydrogen ion in the cells^22^. Other studies showed a significant reduction in the development of chemically induced cancer in animals treated with hesperidin. For example, frequency of oral (tongue), esophageal (food pipe/gullet) and colon carcinoma was reduced by 75, 70, and 61%, respectively^23^^-^ 25. In a human study on esophageal cancers, in a xenograft tumor model, it was reported that hesperidin significantly inhibited tumor growth by inducing cell apoptosis detected by TUNEL assay. The authors stated that it can induce cell apoptosis in esophageal cancer cells via the mitochondrial mediated intrinsic pathway through intracellular accumulation of reactive oxygen species^26^.

The relationship between the burn model including esophageal and hesperidin has been investigated in a limited number of experimental studies. Similar to our study, Anayurt et al. showed that hesperidin was effective in reducing macroscopic and microscopic histopathological damage in a corrosive esophageal burn model and in preventing stricture formation, and had positive effects on nutrition in rats with esophageal burns. They concluded that hesperidin is a potent angiogenic factor^27^. Haddadi et al. stated that hesperidin, as a radioprotector, can initiate angiogenesis by VEGF gene induction. They also showed that it can stimulate epithelialization, collagen deposition and increased cell proliferation. Together, they concluded that these changes may accelerate wound healing, particularly radiation-induced skin damage^28^. In another study, Ekim et al. found that topical sunitinib-hesperidin was more effective than the combination of sunitinib and sunitinib-doxycycline alone in the treatment of corneal neovascularization. They suggested that the combination of sunitinib and hesperidin could be a promising treatment to prevent corneal fibrosis and apoptosis^29^.

MDA is a determinant of lipid peroxidation and provides the emergence of free radical. It can trigger various defense mechanisms with tissue damage caused by the production of reactive oxygen species. One of the primary defense mechanisms is GSH peroxidase. GSH is among the important components of intracellular protective mechanisms against a variety of harmful stimuli, including oxidative stress. MPO activity is an enzyme that explains the presence of neutrophils^30^^,^ 31. Gupta et al. showed that after skin injury, levels of MDA were highly increased while level of GSH peroxidase was decreased when compared to control group^32^. Another study of rat skin excision showed that levels of MDA was increased after skin trauma^33^. Sungkar et al. studied the level of MDA in wounded skin of rats. The authors found that serum level of MDA was increased compared to control group in all wounded animals^34^. In our study, MDA and MPO level were increased and GSH content was decreased after burn injury in Burn group compared to Control group. Administration of hesperidin lowered MDA and MPO level and elevated GSH content in Burn+Hesperidin group compared to Burn group (Table 1).

Burn injury causes many pathological changes in tissues; however, severity can be varied based on type of tissue, exposure time, type of agent, amount of substance, etc. Common histopathological changes are inflammation, tissue damage, leukocyte infiltration, and collagen deposition^35^. In our study, the Control group showed regular esophageal histology. In Burn group, degenerated epithelial cells with nuclei loss, apoptosis, disrupted basement membrane, intense leukocyte infiltration and vascular dilatation and congestion, and degeneration in the muscle layer were observed. Hesperidin treatment reversed these pathologies in the Burn+Hesperidin group (Fig. 1c, H&E staining). Histological scores of epithelialization, collagen formation, and neovascularization were increased in Burn+Hesperidin group after hesperidin administration compared to the Burn group (Table 1).

The cell proliferation antigen Ki-67 is constitutively expressed in dividing mammalian cells. Therefore, it is widely used as a cell proliferation marker^36^. Ki-67 is generally a necessary protein for cell proliferation. Ki-67 index is used for diagnosis, prognostic, and predictive tool in the fields of pathology. Immunohistochemical scoring of Ki-67 is a gold standard for prognosis of many diseases^37^. In one study, injection of antisense oligonucleotides that block Ki-67 expression was found to inhibit cell proliferation^38^. Another study showed that intact Ki-67 expression levels are required for normal proliferation rates in various cell lines^39^. Gerlach et al. studied role of Ki-67 in the rat liver regeneration after partial hepatectomy. The authors found that ki-67 level was dramatically higher in hepatectomized rats compared to control rats after hepatectomy^40^. Farhangkhoee et al. evaluated Ki-67 level for an index of integument viability after burn injury. The authors found that increasing thermal injury affects the Ki-67 level and suggested that level histologically could be used as index to analyze burn depth^41^. In another study, proliferative activity of epithelial cells was analyzed by Ki-67 expression. The authors found that Ki-67 activity was higher in periphery of wound area, hair follicles, and in the endothelial cells^42^. In our study, Ki-67 expression was mainly negative in esophageal tissues in Control group. After burn injury, the Ki-67 expression was highest in Burn group; however, hesperidin treatment lowered Ki-67 expression by alleviating tissue injury in the Burn+Hesperidin group (Fig. 1f, Ki-67 immune staining).

Caspases are cysteine proteases that are involved in cell death. They play very important roles in embryonic development and cell homeostasis. Caspase-3, on the other hand, belongs to the effector caspase group and causes apoptotic cell morphology by degrading the relevant proteins in the cell that will undergo apoptosis^43^. Du et al. showed caspase-3 in wound healing and investigate its role to determine the wound age. They found that caspase-3 was detectable in polymorph mononuclear cells and fibroblastic cells. They also stated that caspase-3 expression was low at initial phases of wound healing but then increased. The authors suggest that caspase-3 plays a role in apoptotic induction of neutrophil, macrophage, and fibroblast, and should be used as a marker for wound age^44^. In an in vitro study on scar tissue, caspase-3 level was immunohistochemically analyzed to show apoptosis in wound healing. The study revealed that caspase-3 proteolytic activities were increased in scar tissues, leading to apoptosis of fibroblasts^45^. Akar et al. evaluated the level of caspase-3 to estimate wound age in rat skin. The authors found that caspase-3 was significantly increased in polymorphonuclear and inflammatory mononuclear cells, and caspase-3 level was time dependent to evaluate apoptosis^46^. Fawzy et al. investigated caspase-3 expression during wound healing of contused skeletal muscle in rats. The authors record that caspase-3 expression was increased in the periphery of the contused and the regenerated muscles. They suggested that immune activity of caspase-3 may be an important marker of muscle apoptosis^47^. In our study, caspase-3 expression was mainly negative in esophageal tissues in the Control group. After burn injury, the expression was highest in the Burn group; however, hesperidin treatment lowered caspase-3 expression by preventing apoptotic pathway in the Burn+Hesperidin group (Fig. 1i, caspase-3 immune staining).

## Conclusion

Hesperidin, as a potent antioxidant, showed an anti-inflammatory effect on the increased Ki-67 signal after burns. It has been observed that hesperidin causes the slowdown of caspase-3 activity by affecting the accelerated apoptotic process due to inflammation. Since there are very limited articles on the relationship between esophageal burn induction and hesperidin, we think that hesperidin dosage and application methods can be developed as an alternative treatment for esophageal burn healing and treatment.

## Data Availability

All generated data presented in this study.
